# An overview of avian influenza in the context of the Australian commercial poultry industry

**DOI:** 10.1016/j.onehlt.2020.100139

**Published:** 2020-05-11

**Authors:** Angela Scott, Marta Hernandez-Jover, Peter Groves, Jenny-Ann Toribio

**Affiliations:** a Primary Industries and Regions South Australia, 33 Flemington St, Glenside 5065, SA, Australia; b Graham Centre for Agricultural Innovation, Pugsley Place, Charles Sturt University, NSW 2650, Australia; c The University of Sydney, c15/335 Werombi Rd, Camden, NSW 2570, Australia

**Keywords:** Australia, Avian influenza, Chicken, Poultry, Risk

## Abstract

From 1976 Australia has experienced seven highly pathogenic avian influenza (HPAI) outbreaks in poultry farms and there have been a total of 16 confirmed low pathogenic avian influenza (LPAI) cases in poultry in Australia at the time of writing. This paper describes all past LPAI and HPAI detections in Australian poultry and reviews avian influenza risk in the Australian commercial chicken industry. The factors that influence this risk are also discussed; notably the nomadic nature of Australian waterfowl, the increasing demand of free range poultry egg and meat production in Australia, and biosecurity practices implemented across farms including farm separations.

## Highlights

• Australia has experienced seven highly pathogenic avian influenza (HPAI) outbreaks in poultry farms
• There have been 16 confirmed low pathogenic avian influenza (LPAI) cases in poultry in Australia at the time of writing
• Australian waterfowl are nomadic in nature
• There is increasing demand of free range poultry production in Australia
• Mathematical models for avian influenza risk in Australia have been reviewed

## 1 Introduction

Avian influenza virus (AIV) is a significant viral pathogen of birds and is a potential zoonosis [51]. It is a RNA virus and is therefore prone to mutations, reassortments and recombinations [43]. This has enabled numerous conversions of low pathogenic avian influenza (LPAI) virus subtypes of H5 or H7 to high pathogenic avian influenza (HPAI) virus [4,41,51]. Birds in the taxonomical orders Anseriformes and Charadriiformes, known as waterfowl and shorebirds respectively, constitute the largest natural reservoir of AIV [34]. For the purpose of this manuscript, ‘poultry’ refers to domestic ground-dwelling birds raised for egg and meat production, such as chickens, ducks and turkeys. LPAI virus introduction from wild birds, leading to subsequent establishment of LPAI virus in gallinaceous poultry, is where most conversions of LPAI to HPAI virus has occurred [51]. HPAI virus from gallinaceous poultry has also consequently been introduced to various other taxonomic groups of wild bird populations such as those in the orders Falconiformes and Passeriformes, leading to further spread and disease, including deaths in these wild bird populations [27]. Globally, disease from AIV, especially HPAI, has caused billions of bird deaths with substantial impacts to poultry industries as well as hundreds of human deaths [[27], [28], [29]]. The human pandemic potential of AIV, that can occur once the virus obtains the ability of human-to-human transmission, is a significant public health concern [56]. The prevention of LPAI virus introduction from wild waterfowl to domestic poultry therefore not only prevents the occurrence of HPAI in poultry industries and subsequent wildlife, but is also a critical step in preventing an AIV-origin pandemic in the human population [51].

There are several factors that influence AIV outbreak risk in Australia. These include the presence of unique Australian viral lineages of LPAI virus in reservoir waterfowl populations [36]. Australian waterfowl are also generally non-migratory and nomadic [34,36]. There has also been a recent, large expansion of the Australian poultry industry due to product demand, where free range production in particular has become a popular choice among retailers and consumers ([9], [12]). This paper reviews AIV in the Australian context including a list all LPAI and HPAI events that have occurred in Australian poultry thus far which has not been documented in a single publication to date. This paper also reviews AIV risk assessments in the Australian commercial chicken industry.

## 2 Australian wild water bird movements and the Australian commercial chicken industry in the context of AIV

Australian waterfowl movements are nomadic; they are in response to rainfall and the presence of waterbodies and other resources [23]. Such movements of Australian waterfowl is markedly different from waterfowl movements in the northern hemisphere, which undergo annual long-distance migrations following specific flight-paths over several continents [17,23]. Few Australian waterfowl such as the wandering whistling duck (*Dendrocygna arcuata*) extend their distances to the Australo-Papuan region where travel from northern Australia to Asia occurs. Such movements are usually confined southeast of the Wallace line; a natural faunal boundary delineating Asian and Australasian flora and fauna that runs through Indonesia between Borneo and Sulawesi and through the Lombok Strait between Bali and Lombok [23,53,54]. In contrast to Australian waterfowl, shorebirds found in Australia do undergo annual long-distance migrations over several continents, such as via the East-Asian Australian flyway [23].

Chicken is now the most consumed meat in Australia, surpassing beef, lamb and pork at 48.6 kg/person/year in 2019 ([10]). Egg consumption in Australia has also increased from under 230 eggs per capita in 2015 to 245 egg per capita in 2018 ([11], [12]). The number of meat chickens greatly surpasses the number of layer chickens on the ground at any point in time in Australia; a typical meat chicken farm houses approximately 240,000 meat chickens at any one time where there are roughly 800 contract grower farms in Australia. Therefore, there is approximately 192 million meat chickens in Australia at any point in time compared with 20 million layer chickens [10,12]. In recent years, there has been an increase in consumer demand for free range chicken products due to the belief that free range production provides better welfare for the bird and produces a higher quality product. This has lead to an increase of free range Australian chicken meat production from 15% in 2011 to 20% in [9,32], and a grocery market share volume of free range Australian eggs from 39% in 2015 to 45% in 2019 of which now surpasses cage egg volumes [11,12].

The increase in free range production raises concerns due to the increased potential contact between wild birds and domestic poultry and the subsequent introduction of pathogens such as AIV [45,51,52]. Vegetative range areas and dams on free range farms can provide permanent residence for nomadic Australian waterfowl on farms and therefore a constant source of AIV infection to poultry ([46, 52, 53]). AIV infection dynamics in Australian waterfowl are also vastly different to those in the northern hemisphere, and are largely influenced by rainfall in Australia [30]. It was found that the risk of incursion of exotic AIV from shorebirds to Australian poultry was more likely to occur via the introduction into nomadic waterfowl populations initially rather than directly from migratory shorebirds, through mixing of the two wild bird populations in common areas such as shoreline and wetland environments [25]. Migratory shorebirds have restricted inland movement and therefore do not come into close proximity to Australian poultry farms [23,25]. A study that conducted wildlife camera trapping on Australian commercial chicken farms identified only one visit of a Charadriiformes bird (a masked lapwing) compared to six Anseriformes bird visits [44].

## 3 Past LPAI and HPAI detections in Australian wild birds and poultry

During a five-year period of risk-based surveillance from 2007 to 2012, the overall proportion of birds in Australia that tested positive for LPAI virus via PCR was 1.9% [34]. The surveillance demonstrated that Anseriformes and Charadriiformes were the bird orders most commonly infected at 2.5% and 0.6% respectively, and a variety of subtypes including H7 were detected [34,36]. HPAI has never been detected in Australian wild birds except in a Eurasian starling (*Sterna* vulgaris) trapped in a HPAI infected poultry shed in 1985 (Table 1); infection in this instance was transmitted from poultry to the wild bird rather than the wild bird being the source of infection for the poultry [15].

**Table 1:** Descriptive characteristics of the seven HPAI outbreaks in Australia from 1976 to 2013.

Year	Month	Subtype	Location of outbreak	Number of affected farms	Description of farms	Cost of eradication (AU$)	Flock size	References
1976	01	H7N7	Keysborough, Victoria	2	Index farm combined conventional chicken meat and caged layer chicken farm. Detection of LPAI also in adjacent free range duck farm (Table 2).	220,000	25,000 layer chickens & 17,000 meat chickens	[16]; [50]
							16,000 ducks	
1985	05	H7N7	Bendigo, Victoria	1	Combined layer chickens, meat chickens and meat chicken breeders on one farm.	2 million	120,000 chickens	[15]; [50]; [55]
1992	07	H7N3	Bendigo, Victoria	2	Index farm a chicken meat breeder farm. Serological infection of AI also in neighbouring duck farm depopulated (Table 2).	1.35 million	17,000 chickens	[31]; [50]; [55]
							6000 ducks	
1994	12	H7N3	Lowood, Queensland	1	Multi-age layer chicken farm.	420,000	22,000 chickens	[24]; [50]
1997	11/12	H7N4	Tamworth, NSW	3	Index farm chicken meat breeder farm. Another chicken meat breeder farm south of index farm infected. Meat emu farm also infected.	4.45 million	128,000 chickens	[48]; [50]
							32,000 chickens	
							260 emus	
2012	11	H7N7	Maitland, NSW	1	Semi-free range layer chicken farm.	465,000	50,000 chickens	[18]; [20]; [38]
2013	10	H7N2	Young, NSW	2	Index farm combined free-range and caged chicken layer farm. Caged layer chicken farm also infected.	3.57 million	160,000 chickens	[18]; [19]
							275,000 chickens	

In Australia, LPAI virus of subtypes H5 or H7 are classed as a category 3 emergency animal diseases (EAD) as these subtypes can cause moderate national socio-economic consequences and have the ability to mutate to HPAI virus. As HPAI has the potential to cause very severe production losses and significant impacts on the national economy, it is classed as a category 2 EAD in Australia [2,5]. Australia has experienced seven HPAI outbreaks in poultry farms since 1976 with details presented in Table 1. The definite source of the outbreaks were not identified but in all farms there was opportunity for direct or indirect contact with waterfowl. All HPAI outbreaks have occurred only in the three eastern states of Australia; Victoria (three separate outbreaks), Queensland (one outbreak), and NSW (three separate outbreaks). The outbreaks involved single farms or small clusters of farms with limited spatial spread. All outbreaks involved commercial chicken farms with large flocks and long-lived, sexually mature chickens of either breeder or layer chickentypes. All viruses were of subtype H7 and of Australian lineages (Table 1).

**Fig. 1:**
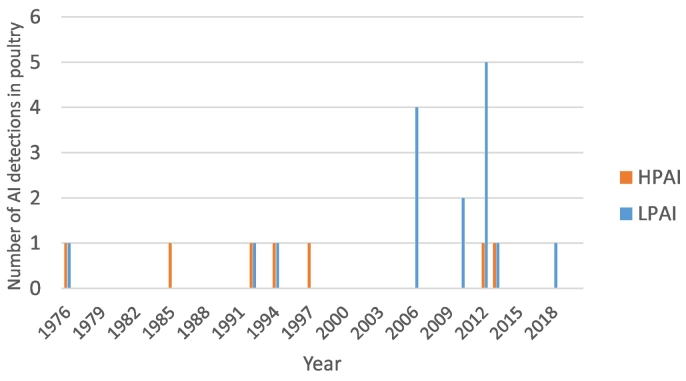
The number of AI detections in domestic poultry in Australia by pathotype (HPAI and LPAI) per year.

Reports of confirmed Australian LPAI cases in poultry are available from 1976. These confirmed cases are the result of passive surveillance (diagnostic submissions), active surveillance (primarily area surveillance during HPAI outbreaks) or incidental findings not associated with a disease or surveillance. At the time of writing, there have been a total of 16 confirmed LPAI cases in poultry in Australia with the latest case occurring in 2018 at the time of writing. Each case represents one farm where there have been positive LPAI virus PCR, virus isolation, or serological evidence of LPAI in poultry on that farm. Clinical signs in poultry in these LPAI events were largely mild, where some cases had no clinical signs apparent. Concurrent bacterial pathogens were associated in all LPAI events with clinically affected ducks. LPAI has never been detected on a single species commercial egg layer enterprise or on poultry farms in South Australia or Northern Territory (Table 2). In 2010, seven abattoir workers reported conjunctivitis and minor upper respiratory tract symptoms after processing clinically normal poultry from the New South Wales farm in which H10N7 occurred. Influenza virus A subtype H10 infection was then detected in two workers [7]. Fig. 1 summarises the number of HPAI and LPAI detections in poultry in Australia per year.

**Table 2:** Descriptive characteristics of the confirmed LPAI reports in poultry in Australia from 1976 to 2018.

Year	State	Subtype	Species on farm	Clinical signs	Farm type	Flock size	References
1976	Victoria	H7N7	Ducks	None	Free range commercial (meat)	16,000	[1]; [6]; [40]; [55]
1992	Victoria	H1, H4, H5, H7, H9	Ducks	None – serological evidence only	Free range commercial (meat)	5700	[1]; [6]; [40]; [55]
1992	Victoria	H3N8	Ducks	Respiratory signs^b^	Barn commercial (breeders & meat)	>40,000	[1]; [6]; [40]; [49]
2006	Tasmania	H5	Chickens and ducks	Chickens: respiratory, mortality (6%) – serological evidence only	Free range non-commercial	300	[1]; [6]; [40]
				Ducks: none			
2006^c^	NSW	H6N4	Chickens	Mortality (0.5%), production drop (10%), gastrointenstinal^b^	Barn commercial (breeders)^e^	>60,000	[1]; [6]; [40]
2006^d^	NSW	H6N4	Ducks	None – serological evidence only	Barn commercial (breeders)	>40,000	[1]; [6]; [40]
2006	QLD	H6N4	Chickens and ducks	Chickens: respiratory, mortalities (mild)	Free range, non-commercial	100	[1]; [6]; [40]
				Ducks^a^: none			
2010^c^	NSW	H10N7	Chickens	Mortalities (mild),	Barn commercial (breeders)^e^	>60,000	[1]; [6]; [40]
				Production drop (15%)			
2010^c^	NSW	H1	Ducks	None – serological evidence only	Barn commercial (breeders)	>40,000	[1]; [6]
2012	Victoria	H5N3	Ducks	Respiratory,	Free range commercial (meat)	24,000	[1]; [6]; [42]
				Musculoskeletal^b^			
2012	NSW	H4N6	Chickens, geese and ducks	Chickens: none	Free range commercial (meat)	2500	[1]; [6]
				Geese: none			
				Ducks^b^: respiratory, mortalities (mild)			
2012	NSW	H9N2	Turkeys	Respiratory, mortalities (24%)	Barn commercial (meat)	40,000	[1]; [6]
2012	NSW	H9N2	Turkeys	None	Barn commercial (meat)	40,000	[1]; [6]
2012	QLD	H10N7	Chickens and ducks	Chickens^a^: none	Free range commercial (layers)	6100	[1]; [6]
				Ducks^b^: respiratory, mortalities (marginal) - serological evidence only			
2013	Western Australia	H5N3	Chickens and ducks	Chickens: none	Free range non-commercial	95	[1]; [42]
				Ducks^a^: none			
2018	QLD	H1N2	Chickens, ducks and guinea fowl	Chickens: none	Free range non-commercial	50	[3]
				Ducks: mortalities (mild)			
				Guinea fowl: mortalities (mild)			

a Virus isolation from respective species occurred.

b Co-infection with bacterial pathogen also diagnosed.

c Number donates the same farm but different incident/year of LPAI detection.

d Number donates the same farm but different incident/year of LPAI detection.

e Farm reported to have had excellent biosecurity.

## 4 Avian influenza risk assessments for the Australian commercial chicken industry

A quantitative exposure assessment estimated that the probability of a first LPAI virus exposure to an Australian commercial chicken farm from a single wild bird present on the farm at any point in time was extremely low. Free range layer farms were the most likely farm type to experience an LPAI virus introduction [46]. However, the assessment demonstrated that changes in the probability of exposure based on the number of wild birds present on a farm at any point in time, the proportion of wild birds on the farm that are waterfowl, and changes in LPAI virus prevalence of waterfowl showed significant changes in LPAI virus introduction risk. It was found that the largest number of exposures occurs when the proportion of waterfowl is increased to 80% and the AIV prevalence increased to 20% [46]. As noted in past HPAI events (Table 1) and by [30], waterfowl may make up a considerable proportion of wild birds on a property during drought or rainfall events. The prevalence of LPAI virus in waterfowl may also increase with population dynamics, such as an increase in immune-naive juvenile waterfowl. Sensitivity analysis reinforced these findings, highlighting the importance of continued AIV surveillance in wild birds in Australia [36,46]. Methods to deter waterfowl from farms are therefore greatly influential in reducing AIV introduction risk. Current methods such as netting ranges and dams are largely cost-prohibitive. Technology that detects and deters waterfowl specifically has been developed, with further refinements necessary to become a cost-effective and widespread tool [8,39].

There was limited spread to other farms in all AIV outbreak detections in Australian poultry, attributable to the rapid stamping out response [2] (Table 1). However, the Australian poultry industries have been assessed as vulnerable to large outbreaks of HPAI. Other countries which have experienced widespread HPAI outbreaks have common with Australia, such as dense farm areas and frequent farm to farm [35]. Equipment was identified as the most likely pathway for the spread of AIV between sheds, and poultry pick up systems and egg trays for spread between farms [47]. The common practice of sharing equipment and vehicles between farms highlights the importance of advocating the concept of shared responsibility to biosecurity, particularly within the Australian egg industry where large variations in farm size and differing levels of biosecurity practice implementation exist [37,45]. Shared responsibility involves the industry, governments, and the broader community to work together across the biosecurity continuum on ‘prevention, emergency preparedness, detection, response, recovery and ongoing management of pests and diseases’ [21,37]. Farm separation distances is also an important aspect to biosecurity on poultry farms to limit the spread of pathogens from farm to farm. In particular, the separation of different species of poultry [22,26]. This is important to limit the potential spread of AI from domestic ducks to chickens, of which the former species may act as a reservoir species and not show clinical signs as recognised worldwide and described in Table 2 [26].

The influence on farm type on AI outbreak risk in the Australian commercial chicken industry was assessed through branching process models. It was found that a 25% change in the proportion of farms in the Australian commercial chicken industry to free range farming would increase the probability of a HPAI outbreak by 6–7%, rising to 12–14% with a 50% change to free range farming [33]. In addition, simply due to the large number of chicken meat farm types in the Australian commercial chicken industry relative to other farm types, chicken meat farm types are hypothesised to experience the most LPAI virus introductions but their depopulation at 5–7 weeks of age mitigated HPAI virus emergence [13,14]. This finding as well as HPAI outbreak history in Australia supports the hypothesis that it is most likely that frequent LPAI virus introductions occur in Australian chicken farms with a low mutation rate, rather than infrequent LPAI virus introductions and a high mutation rate [14]. Although HPAI outbreak risk can increase with more free range poultry production, the branching process models showed that it could be compensated by improvements in biosecurity practice implementation. It was found through modelling that treating drinking water significantly reduces HPAI outbreak risk by 25–28% compared to no water treatment. Halving the presence of wild birds around feed storage areas and inside sheds could reduce HPAI outbreak risk by 16–19% and 23–25% respectively [33].

## 5 Conclusion

All past HPAI outbreaks in poultry in Australia were found to align with stochastic mathematical methods of frequent LPAI virus introductions with low probability of mutation. However, there are significant influences on LPAI virus introduction and HPAI outbreak occurrence risk. The review of risk assessments which used stochastic mathematical models have highlighted the importance of continued AIV wild bird surveillance, and to advocate good biosecurity practices including waterfowl deterrence on farms due to these factors' strong influence on LPAI virus introduction probability. The latter is particularly significant in compensating for the increase in HPAI outbreak risk that will occur from the increased proportion of free range commercial chicken farms in Australia. Further explorations of AIV infection dynamics in the Australian context can be conducted through validation of the models, such as through structured population-based surveillance of commercial meat or layer chickens at slaughter. It is important that ongoing research of AIV in the Australian context is performed to prepare for changes in AIV risk.

## Ethical statement

Not applicable.

## Declaration of Competing Interest

The authors declare that they have no known competing financial interests or personal relationships that could have appeared to influence the work reported in this paper.
